# Progressive sensory ataxia and breast implant rupture, an uncommon presentation of a debated concept: a case report

**DOI:** 10.1186/s12883-022-02894-w

**Published:** 2022-09-24

**Authors:** Sofie Van Assche, Heleen Parmentier, Gaelle Varkas, Isabelle Peene, Sarah Herdewyn

**Affiliations:** 1 Neurology Department, University Hospital Ghent, 9000 Ghent, Belgium; 2 Rheumatology Department, University Hospital Ghent, 9000 Ghent, Belgium

**Keywords:** Ganglionopathy, Sjögren’s syndrome, ASIA, Silicone

## Abstract

**Background:**

Autoimmune Syndrome Induced by Adjuvants (ASIA) is a concept introduced by Shoenfeld to group various disease entities believed to be triggered by an infection, silicone exposure or other external stimuli. A causal link between the use of silicone and the development of autoimmune diseases and lymphoma has been suggested in the past. Sjögren’s Syndrome (SS) is one of the autoimmune diseases that has been postulated as an example of ASIA syndrome. Although typically characterized by sicca, SS can manifest as a ganglionopathy as the primary presenting symptom. To our knowledge, this is the first case report in which a ganglionopathy unveiled an underlying SS in the context of a possible ASIA syndrome.

**Case presentation:**

We describe a case of a 44-year-old woman who developed rapidly progressive sensory loss in the 4 limbs with a walking impairment due to the severe sensory ataxia. After extensive work-up, she was diagnosed with a ganglionopathy as the first symptom of SS, and the concurrent diagnosis of a bilateral breast implant leakage with severe inflammation due to silicone bleeding. After surgical removal of the prostheses and initiation of immunosuppressive therapy, stabilization of symptoms was achieved.

**Conclusion:**

This case report brings to attention the possibility of a sensory ganglionopathy as first and isolated symptom of SS. The occurrence of SS in the setting of ASIA stir up the discussion about the safety of silicone breast implants.

## Background

The Autoimmune/inflammatory Syndrome Induced by Adjuvants (ASIA) is a relatively new concept, introduced in 2011 by Shoenfeld [1]. It is a disease entity consisting of a variety of systemic symptoms. It is thought to have its origin in a triggered immune system due to a certain adjuvant. A possible genetic predisposition is suspected, mainly focusing on the HLA-DRB1, DR5 and DQ2 haplotypes and PTPN22 gene. The systemic symptoms described are divers and often vague: diffuse musculoskeletal pain, fatigue, headache, paresthesia, cognitive impairment, weight loss, unexplained fever, skin abnormalities, hair loss, dry eyes and mouth [2]. However, sometimes a distinctive set of clinical features is observed compatible with known disease entities such as sarcoidosis, Sjögrens’s Syndrome (SS), undifferentiated connective tissue disease [3]. An important feature of the ASIA syndrome is that symptoms tend to improve once the causative agent is eliminated. The finding of autoantibodies is possible, although not necessary. Since the introduction of the concept of ASIA, certain triggers have been suggested: drugs, metals, infections, vaccines,... One of the triggers also frequently mentioned in literature is silicone, a material that has been considered biologically inert, which has justified its broad use in medical devices and for instance in reconstructive surgery with breast implants. The following case report concerning a suspected case of ASIA triggered by silicone breast implant rupture is remarkable both in its initial clinical presentation as in the possible underlying disease mechanism.

## Case presentation

### Case description

A 44-year-old female presented at our emergency department with a rapidly progressive sensory loss of the lower limbs combined with severe balance difficulties.

Her medical history included atopic eczema, gastric banding and plastic surgery for breast augmentation. Both surgeries had taken place almost 20 years ago. There were no relevant findings in her family history. Aside from vitamin B and folic acid, started recently because of the symptoms, she denied taking any prescription drugs.

Three months earlier, her symptoms had started with paresthesia and dysesthesia in her feet and lower part of her legs, more prominent on the right side. The nerve conduction studies (NCS) and electromyography (EMG) were only minimally abnormal with an increased latency of the sural nerve sensory nerve action potential (SNAP). Given her complaints, there was a suspicion of a sensory polyneuropathy. The initial work-up before presentation at our hospital, showed a mild lymphocytosis (3.42; reference value 1–3.2 × 10^3^/mm^3^) and serum folic acid deficiency (2.5; reference value 3.9–26.8 μg/L). Vitamin B12 was normal (369 nmol/L, reference values 197–779 nnmol/L). Vitamin B and folate substitution were started. Contrast-enhanced lumbar and cervical spine magnetic resonance imaging (MRI) did not show any abnormalities. A brain MRI with gadolinium was also normal. Two months after the onset of symptoms, a lumbar puncture was performed, which showed a marginally elevated white blood cell count (7.2; reference value 0–5/μL), normal glucose (52, reference value 40-70 mg/dL), in the absence of protein elevation (74.1; reference value 64-83 g/L). However, there was a raised IgG index (0.97; reference value 0.3–0.77) and three supplementary bands in the cerebrospinal fluid on iso-electrical focusing. Because an underlying autoimmune disease was suspected, erythrocyte sedimentation rate, antinuclear antibodies (ANA) and anti-neutrophil cytoplasmic antibodies (ANCA) were checked. Erythrocyte sedimentation appeared slightly elevated, whereas ANCA and ANA were found to be negative, aside from cytoplasmic staining with AC 18 pattern on indirect immunofluorescence on HEp-2 cells. Vitamin B6 level was elevated (1430 nmol/L, reference values 35–110 nmol/L), but was measured after starting substitution and not-fasting.

Three months after onset, the patient was referred to our tertiary centre for further work-up. By this time, the paresthesia in her distal lower limbs had evolved to a numbness of both legs, chest and back. Since a few weeks her hands were also involved in an asymmetrical and patchy pattern. Meanwhile, she had developed a severe gait instability, especially when walking barefoot or in the dark, resulting in multiple falls. Apart from fluctuating paresthesia in her face (especially in the left lower cheek), there was no other cranial nerve involvement: no diplopia, no dysarthria or dysphagia, no signs of facial palsy.

At first clinical evaluation, she had normal strength in both upper and lower limbs, but global areflexia. She had severe sensory deficits with almost absent vibration sensation, impaired proprioception and absent graphesthesia in the lower limbs. She indicated a sensory level around Th 7–8. There was severe ataxia in both legs, both on heel-to-shin test as on walking. She was unstable, needed support and had to look at her feet, which exhibited a pseudo-athetosis when walking.

### Investigations

The patient was admitted to our neurology department for further work-up. Given the new sensory symptoms in the face and the sensory level, neuroimaging was repeated with contrast-enhanced MRI of the brain and the full spine without signs of central demyelination or peripheral inflammation.

Nerve conduction studies now showed bilateral absent sural nerve SNAPs and undetectable right ulnar nerve SNAP. Right median nerve SNAP was still normal, but right radial and left ulnar SNAP were markedly reduced (with an amplitude of respectively 4 μV and 6 μV, normal values > 11 μV). Motor responses were normal, as well as needle EMG findings and blink reflex. Somatosensory evoked potentials showed a normal N8-latency (at the popliteal fossa), no detectable responses in the lumbar region (possibly constitution-related), an absent cortical response from the right leg and a delayed cortical response from the left leg. In summary, electrophysiological testing revealed a non-length dependent, asymmetric loss of SNAPs, best in keeping with a sensory ganglionopathy.

MRI full spine did not show the dorsal column atrophy that is sometimes seen in the chronic phase of a ganglionopathy [4, 5], but this could well be due to the early timing of the imaging in the disease course.

Laboratory results were negative for paraneoplastic antibodies, anti-ganglioside antibodies, anti-aquaporin 4/anti-neuromyelitis optica (NMO), anti-myelin oligodendrocyte glycoprotein (MOG) and coeliac antibodies. Further, we noted a HbA1C of 5.8%, with normal serum protein electrophoresis and copper levels. Following vitamin levels were normal (i.e. vitamin B1 252 nmol/L reference values 67-200 nmol/L; B12 542 ng/L, reference values 197–771 ng/L; folic acid > 20 μg/L; vitamin E 1.4 mg/dL, reference values 0.5–1.8 mg/dL), as well as homocysteine and methylmalonic acid. Vitamin B6 was somewhat elevated (vitamin B6 585 nmol/L, reference values 35-110 nmol/L), but likely not-fasting. These levels were measured while already taking vitamin supplements (combination of folic acid and vitamin B1-B2-B6-B12). Serology for Human Immunodeficiency Virus (HIV), Hepatitis (A/B/C/E), Herpes Simplex, Varicella Zoster, Borrelia/Lyme, and Syphilis were all negative. Auto-immune screening for rheumatoid factor, anti-cyclic citrullinated peptide, ANA, ANCA, cryoglobulinemia and complement levels revealed atypical ANCA (4+) and ANA (2+) with further extractable nuclear antigen (ENA) differentiation positive for anti-Ro52 antibodies.

**Fig. 1:**
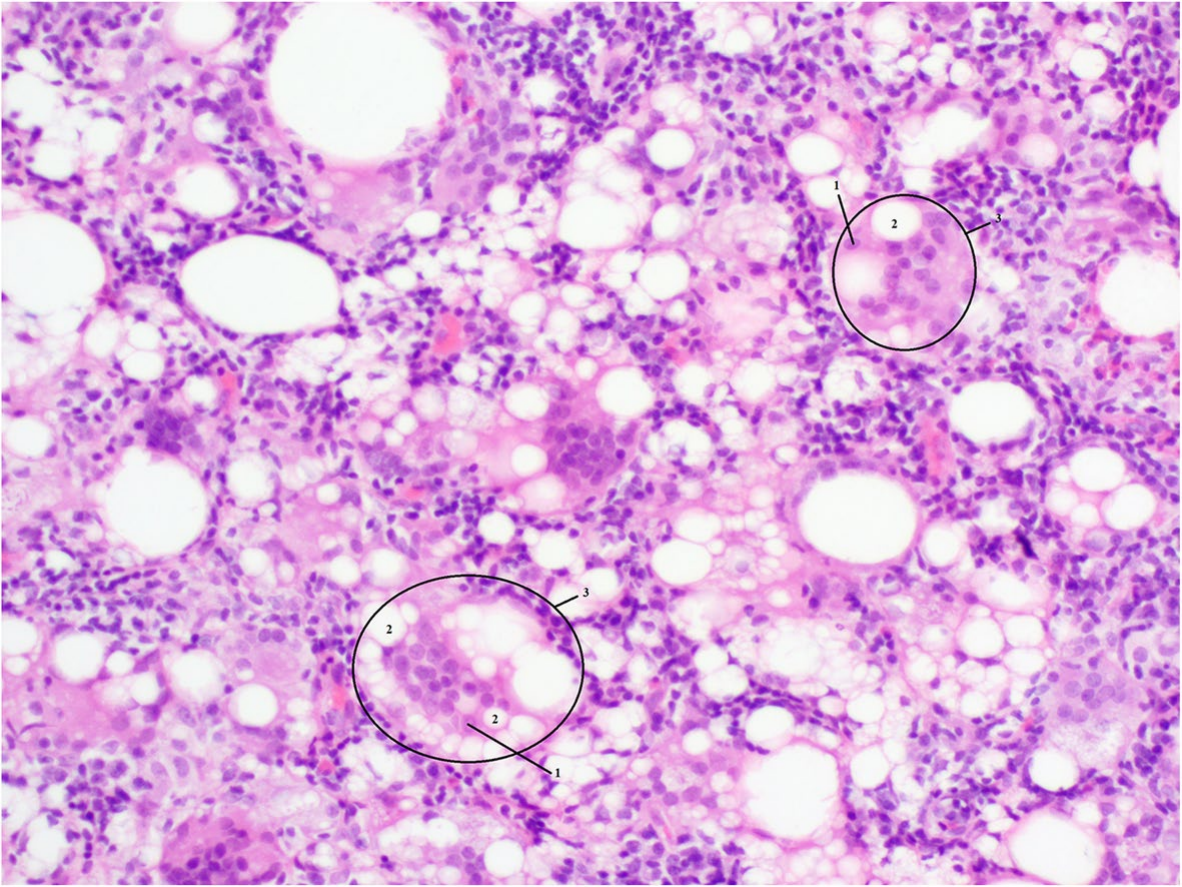
Biopsy of lymph node. Lymph node biopsy with foreign body giant cell reaction and the presence of macrophages containing clear, refractile, nonpolarizable material within cytoplasmic vacuoles. Hematoxylin&Eosin (HE) staining; original magnification 200x. 1 nucleus of one macrophage, 2 silicone particle, 3 cluster of macrophages surrounding silicone particles. Technical information: Obtained using Olympus BX53 microscope; Olympus PlanC 4x/0.10, 10x/0.25, 20x/0.40, 40x/0.65 objective lenses; Olympus 10x/22 oculars; Toupcam industrial digital camera UCMOSO5100KPA 6.1 MP 1/2.5″ APTINA CMOS SENSOR P/N: TP605100A UCMOS Touptek Photonics, Olympus U-TV0.5XC-3; serial number filter 7E44103 201,705; Toupview software by Touptek. No downstream processing or averaging was applied

A clinical evaluation by the rheumatologists was requested, given the anti-Ro52 antibodies and suspicion of SS. There were no signs suggestive of connective tissue disease, e.g. no Raynaud phenomenon, arthralgia/myalgia, glandular swelling, sicca (xerostomia, xerophthalmia), cough, dyspnoea, nor signs of autonomic dysfunction. Ultrasonography revealed heterogenicity of the submandibular gland. Subsequent biopsy of the minor salivary glands confirmed a focus score of more than one, a histopathological finding compatible with SS (Fig. 1). Additionally, performing a Schirmer’s test could have been useful, though the patient was not open to this.

**Fig. 2:**
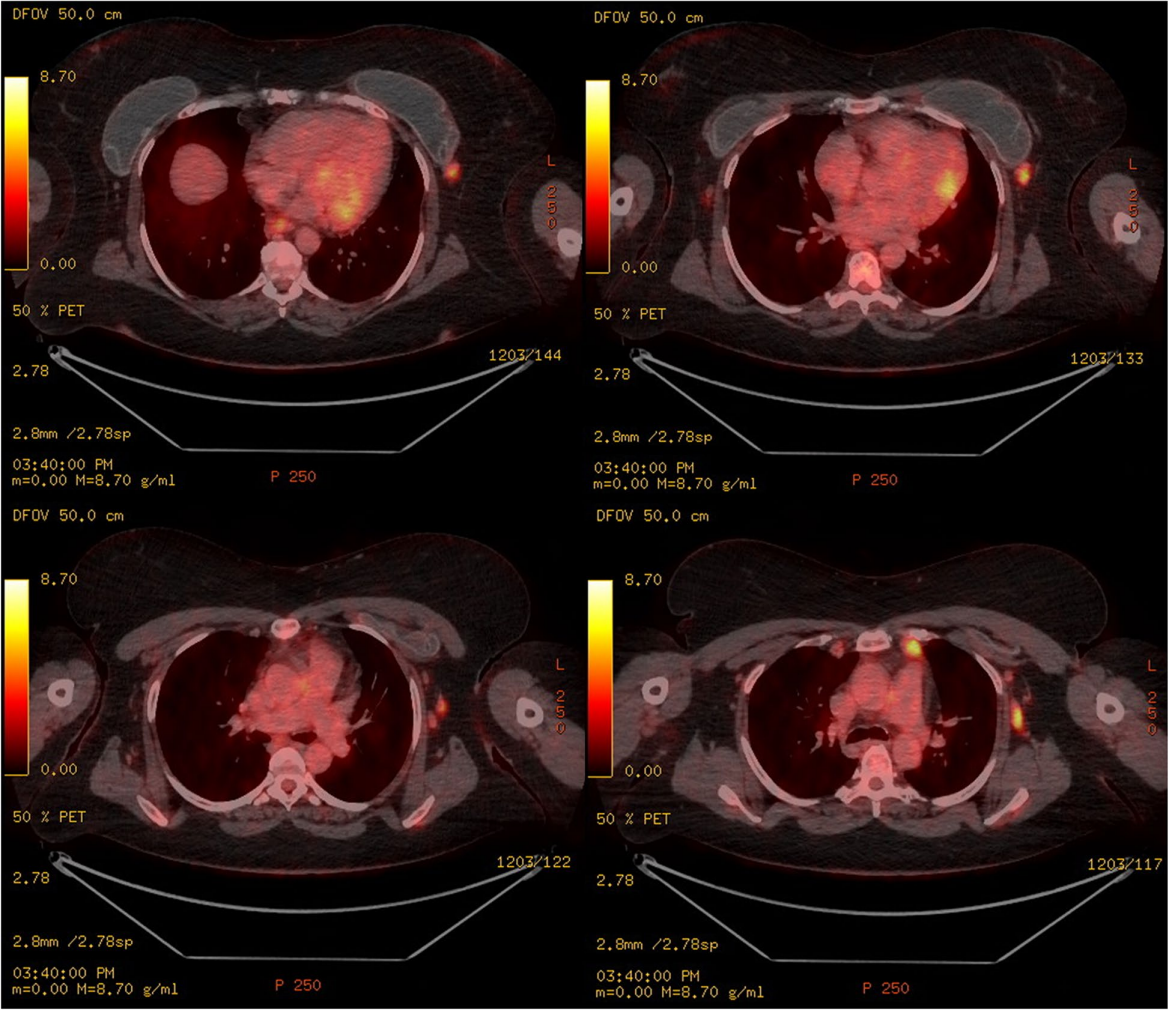
PET-CT imaging. PET-CT displaying hypermetabolic, enlarged lymph nodes, predominantly at the left side. Heterogenic content of the subpectoral breast prosthesis, suggestive of rupture

**Fig. 3:**
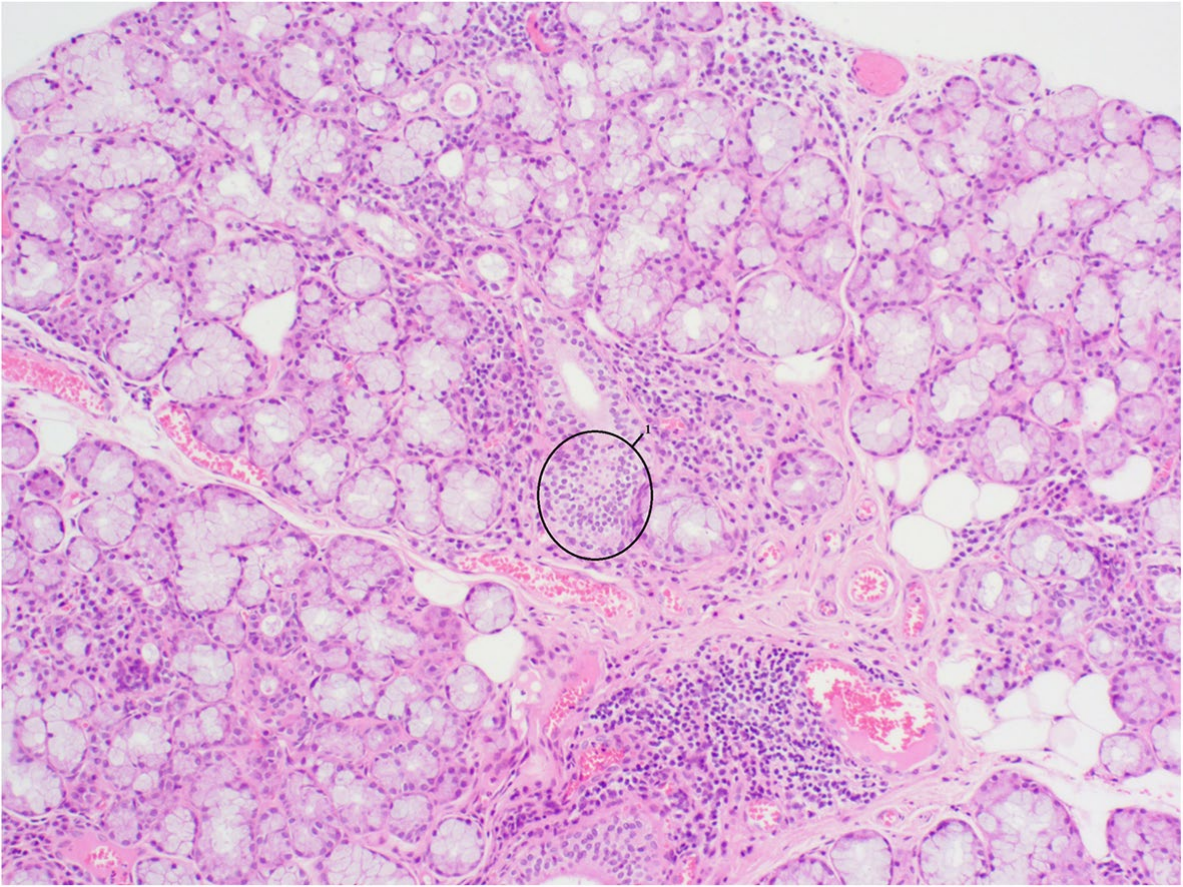
Biopsy of minor salivary gland. Biopsy of minor salivary gland tissue. Hematoxylin&Eosin (HE) staining; original magnification 200x. 1 lymphocytic focus of > 50 lymphocytes. Technical information: Obtained using Olympus BX53 microscope; Olympus PlanC 4x/0.10, 10x/0.25, 20x/0.40, 40x/0.65 objective lenses; Olympus 10x/22 oculars; Toupcam industrial digital camera UCMOSO5100KPA 6.1 MP 1/2.5″ APTINA CMOS SENSOR P/N: TP605100A UCMOS Touptek Photonics, Olympus U-TV0.5XC-3; serial number filter 7E44103 201,705; Toupview software by Touptek. No downstream processing or averaging was applied

A full body positron emission tomography-computed tomography (PET-CT) showed enlarged lymph nodes extending from the left axilla to the left side of the left breast prosthesis and alongside the left thoracic internal artery (Fig. 2). Additional dedicated ultrasonography confirmed leakage of silicone of both breast implants and enlarged lymph nodes compatible with silicone lymphadenopathy. Biopsy confirmed lymphoid tissue surrounding silicone material and could exclude a lymphoma (Fig. 3).

### Treatment

The ruptured breast implants were resected 1 month after the presentation at our emergency department. A few days later, our patient reported an improvement of her general well-being and a stagnation of the - until then - progressive sensory complaints. The reported paresthesia in her face disappeared before any additional therapy was started, but the overall clinical picture remained stable over the next month. Vitamin B supplementation was stopped. After exclusion of malignancies in the resected breast tissue, oral corticosteroid therapy was initiated (at a dose of 60 mg prednisolone a day) in association with hydroxychloroquine 200 mg two times daily and methotrexate 15 mg once a week with folic acid 1 mg daily for 6 days a week. Preventive measures with a proton-pump inhibitor, calcium supplement, Vit D, trimethoprim/sulphametoxazole 160 mg/800 mg (3 times a week) and weekly bisphosphonates were added to her therapy. Steroids were tapered by 10 mg a month. At 3 months follow-up, she did not report new improvement of her symptoms. However, repeated NCS did show a clear amelioration with presence of sural and ulnar SNAP and a normalised radial SNAP. Further follow-up is necessary to show whether there will be clinical improvement.

## Discussion and conclusions

We report a case of ganglionopathy as a first manifestation of SS, in a broader context of ASIA syndrome. The latter highlights the coexistence of some syndromes and concepts classified under the title of Autoimmune/inflammatory Syndrome Induced by Adjuvants (ASIA) [1]. We briefly discuss the different disease entities and try to link them as a whole.

*Ganglionopathy* is typically characterised by an asymmetric, non-length dependent sensory deficit with severe ataxia and areflexia at clinical examination [6]. Facial involvement, autonomic dysfunction, cerebrospinal fluid (CSF) pleocytosis and oligoclonal bands have been reported [7]. Proposed diagnostic criteria by Camdessanché et al. are shown in Table 1. Our patient had a score of 12.7 (Aa, Ab, Ac, Ad, Ae) and therefore fulfilled the criteria for probable ganglionopathy (Ba and Bbv).

**Table 1:** Criteria sensory neuronopathy (SN) as proposed by Camdessanché et al. [7]

**A—Possible SN** if score > 6,5	
(a) Ataxia in the lower or upper limbs at onset or full development	3.1
(b) Asymmetrical distribution of sensory loss at onset or full development	1.7
(c) Sensory loss not restricted to the lower limbs at full development	2.0
(d) At least 1 SNAP absent or 3 SNAP < 30% of the lower limit of normal in the upper limbs, entrapment neuropathies excluded	2.8
(e) < 2 nerves with abnormal motor nerve conduction studies in the lower limbs	3.1
**B—Probable SN** if score > 6,5 AND (a) AND ((b) or (c))	
(a) The initial workup does not show biochemical or electrophysiological findings excluding SN	
(b) The patient has one of the following: i. diagnosis of cancer within 5 years ii. cisplatin treatment iii. Detection of onconeural AB iv. diagnosis of an HIV infection v. Sjögren’s syndrome	
(c) MRI shows T2-hyperintensity in the posterior column of the spinal cord	
**C—Definite SN** pathological demonstration of dorsal root ganglia degeneration trough biopsy	

*Primary Sjögren’s syndrome* (pSS) is known for its glandular tropism, but the typical sicca symptoms can be accompanied or even preceded by many different extra-glandular manifestations as reflected by the European Sjögren’s Syndrome activity index (ESSDAI) such as constitutional symptoms, lymphadenopathy, articular involvement, cutaneous involvement, pulmonary involvement, renal involvement, peripheral/central nervous system involvement and/or haematological involvement [8–12]. The diagnosis of SS is ultimately made clinically, supported by the 2016 ACR-EULAR criteria, as presented in Table 2 [13, 14].

**Table 2:** ACR-EULAR criteria for Sjögren’s syndrome as proposed by Ramos-Casals M, Brito-Zerón P, Bombardieri S, et al. [13]

Diagnosis Sjögren’s syndrome if A and C present, B excluded	
A— Inclusion criteria at least one of the following	
(a) oral or ocular dryness based on questioning	
(b) suspicion of SS with at least 1 positive item on ESSDAI^A^	
B— Exclusion criteria	
(a) head or neck irradiation	
(b) AIDS, active hepatitis C	
(c) sarcoidosis, amyloidosis, IgG4-related disease	
(d) graft versus host disease	
C— Score ≥ 4	
(a) labial salivary gland with focal lymphocyte sialadenitis and focus score ≥ 1	3
(b) anti-SSA (Ro) antibodies	3
(c) ocular staining score^B^ ≥ 5, or Van Bijsterveld score^C^ ≥ 4 in at least one eye	1
(d) Schirmer’s test ≤5 mm in 5 minutes in at least one eye	1
(e) unstimulated whole saliva^D^ flow rate ≤ 0,1 ml/min	1

A ESSDAI: The EULAR Sjögren’s Syndome Disease Activity Index.

B described in Whitcher et al. [15]

C described in Van bijsterveld et al. [16]

D described in Navazesh and Kumar [17]

Neurological involvement is seen in 20% of the patients with primary SS (pSS), with peripheral nervous system symptoms in 5–20% and central nervous system symptoms in 1–5% [9]. Sensory neuronopathy (SN) or ganglionopathy is considered a rare manifestation of pSS, but can be the first presentation [18]. Pereira et al. described a group of 13 patients with a SN due to pSS of whom 11 patients had a SN at first presentation [19]. Although rare, pSS is the most common underlying autoimmune disease in a patient presenting with sensory ganglionopathy, as was true for our patient.

The debated concept of an *Autoimmune/inflammatory Syndrome Induced by Adjuvants (ASIA*) was introduced in 2011 by Shoenfeld to group different medical conditions with the same presumed pathophysiological background, being siliconosis, Gulf War Syndrome, macrophage myophaciitis, sick building syndrome and post-vaccination phenomena. The concept of the disease entity is based on the underlying idea that exposure to certain materials (adjuvants) can induce an immunological response in susceptible individuals. The predisposition is thought to be genetically linked to certain HLA haplotypes (HLA-DRB1, DR5; DQ2 and PTPN22 gene) [1, 20, 21]. The most reported symptoms for ASIA are rather vague, but distinct syndromes have been recognised. The diagnostic criteria proposed are listed in Table 3. Our patient fulfilled all the major criteria.

**Table 3:** Criteria ASIA syndrome as proposed by Shoenfeld et al. [1, 3]

**A—Major criteria**	
(a) exposure to an external stimulus prior to clinical manifestation (e.g. infection, vaccine, silicone, ...)	
(b) typical clinical manifestations I. myalgia, myositis or muscle weakness II. arthralgia and/or arthritis III. chronic fatigue, sleep disturbances IV. neurological manifestations V. cognitive impairment VI. pyrexia, dry mouth	
(c) removal of trigger induces improvement	
**B— Minor criteria**	
(a) autoantibodies or antibodies directed against the adjuvant	
(b) specific HLA (e.g. HLA DRB1, HLA DQB1)	
(c) evolvement of an autoimmune disease (e.g. SSc)	

Throughout the literature there appears to be a consensus that having either two major criteria, or one major and two minor criteria, is sufficient for a diagnosis of ASIA [21, 22]

The first silicone breast implant dated from the early 60s. Silicone was mistakenly thought to be inert in the human body. Already in 1964 Mioshy et al. described the occurrence of connective tissue disease after cosmetic surgery. This was further illustrated by animal models showing an inflammatory response around injection sites of silicone with attraction of leukocytes (macrophages, plasma cells, eosinophils), fibroblasts and formation of fibrous adhesions and histiocytic granulomas [23]. Also, more recently, antibody production after silicone instillation in genetically predisposed mice has been demonstrated [24, 25]*.* This inflammatory response has also been shown in humans with axillary lymphadenopathy, with formation of fibrous capsules and silicone granulomas after leakage of silicone breast implantation and/or dermal injections of silicone [23, 26, 27]. Case reports and cohort studies suggest that apart from a local inflammatory response (directly adjacent to the breast implant and in lymphoid tissue of the axilla and the thoracic chain), also a more general systemic reaction to silicone breast implantation is triggered [2, 28, 29].

More specifically, different case reports and case series report development of SS following silicone exposure [30, 31]. Although still debated, epidemiological studies have shown an increased risk of developing auto-immune disease after silicone breast implantation. For instance, for SS, an increased odds ratio of 1.58 was seen in women with silicone breast implants compared to controls [32]. A large systematic review could not determine a causal relationship between silicone breast implants and connective tissue disease, but the higher prevalence of SS in patients with silicone breast implantation was confirmed [23, 33]*.*

It is important to mention that a lymphoma was excluded by biopsy, given the fact that the FDA warned in 2019 for the higher risk of development of lymphoma in patients with breast augmentation, while the risk of lymphoma is also increased in SS [17] .

In this case presentation, the history of gastric banding is important, given the possibility of vitamin deficiencies (mostly B12) after bariatric surgery [34, 35]. However, in our patient Vitamin B12 levels have always been normal. Serum folic acid level was slightly low, but with normal homocysteine and methylmalonic acid. Furthermore, a vitamin B12 and folic acid deficiency are not known to cause a true sensory neuronopathy. A contributing factor of a possible B1 deficiency was not totally excluded, as she was seen by us after starting vitamin B substitution. However, thiamine deficiency presents with a length dependent polyneuropathy and our patient had a non-length dependent process i.e. ganglionopathy. Vitamin B6 toxicity can cause a ganglionopathy, but the blood samples to measure this were taken while on substitution therapy and (probably) not-fasting. Additionally, substitution was started after the first complaints and the symptoms had already improved before withholding the vitamin supplements. Thus, in this patient, the gastric banding and substitution therapy, are highly unlikely to explain the clinical picture.

In conclusion, we encountered a rare clinical presentation of SS in which the patient developed a sensory ganglionopathy without other typical symptoms of SS. In the work-up towards her diagnosis we found leakage of silicone breast implant causing a local inflammatory response both on imaging and biopsy. After resection of the prosthesis, she reported a stabilization to slight improvement of her symptoms. The timing of diagnosis of both medical problems, the spontaneous improvement after resection and the existing evidence from literature underscore a causal relationship between the silicone leakage and the development of SS in this patient. The whole picture can well be an example of an ASIA syndrome.

The occurrence of SS in the setting of ASIA stir up the discussion about the safety of silicone breast implants. Removal of the breast implants in our patient in combination with immunosuppressive therapy halted the progression of the ganglionopathy with mild perceived improvement and a clear favourable evolution on NCS. Further evolution is to be awaited, but it is known that improvement of a SN can take many months to years and is often incomplete [6].

Development of SS following silicone exposure has been reported previously. However, to our knowledge, this is the first case report where a sensory ganglionopathy was the initial presentation of an ASIA syndrome.

## Data Availability

Data sharing is not applicable to this article as no datasets were generated or analyzed during the current study.

## Abbreviations

ASIA: Autoimmune/inflammatory Syndrome Induced by Adjuvants
SS: Sjögren’s Syndrome
pSS: Primary Sjögren’s Syndrome
ESSDAI: European Sjögren’s Syndrome Activity Index
NCS: Nerve Conduction Studies
EMG: Electromyography
SNAP: Sensory Nerve Action Potential
ANA: Antinuclear Antibodies
ANCA: Anti-Neutrophil Cytoplasmic Antibodies
MRI: Magnetic Resonance Imaging
IgG: Immunoglobulin G
Anti-NMO: Anti-Neuromyelitis Optica
Anti-MOG: Anti-Myelin Oligodendrocyte Glycoprotein
ENA: Extractable Nuclear Antigen
PET-CT: Positron Emission Tomography – Computed Tomography
CSF: Cerebrospinal Fluid
SN: Sensory Neuronopathy
